# Dual effect of fetal bovine serum on early development depends on stage-specific reactive oxygen species demands in pigs

**DOI:** 10.1371/journal.pone.0175427

**Published:** 2017-04-13

**Authors:** Seong-Eun Mun, Bo-Woong Sim, Seung-Bin Yoon, Pil-Soo Jeong, Hae-Jun Yang, Seon-A Choi, Young-Ho Park, Young-Hyun Kim, Philyong Kang, Kang-Jin Jeong, Youngjeon Lee, Yeung Bae Jin, Bong-Seok Song, Ji-Su Kim, Jae-Won Huh, Sang-Rae Lee, Young-Kuk Choo, Sun-Uk Kim, Kyu-Tae Chang

**Affiliations:** 1 National Primate Research Center, Korea Research Institute of Bioscience and Biotechnology, Chungcheongbuk-do, Republic of Korea; 2 Futuristic Animal Resource & Research Center, Korea Research Institute of Bioscience and Biotechnology, Chungcheongbuk-do, Republic of Korea; 3 Department of Biological Science, College of Natural Sciences, Wonkwang University, Jeollabuk-do, Republic of Korea; 4 Department of Functional Genomics, University of Science and Technology, Daejeon, Republic of Korea; Peking University Third Hospital, CHINA

## Abstract

Despite the application of numerous supplements to improve *in vitro* culture (IVC) conditions of mammalian cells, studies regarding the effect of fetal bovine serum (FBS) on mammalian early embryogenesis, particularly in relation to redox homeostasis, are lacking. Herein, we demonstrated that early development of *in vitro*-produced (IVP) porcine embryos highly depends on the combination of FBS supplementation timing and embryonic reactive oxygen species (ROS) requirements. Interestingly, FBS significantly reduced intracellular ROS levels in parthenogenetically activated (PA) embryos regardless of the developmental stage. However, the beneficial effect of FBS on early embryogenesis was found only during the late phase (IVC 4–6 days) treatment group. In particular, developmental competence parameters, such as blastocyst formation rate, cellular survival, total cell number and trophectoderm proportion, were markedly increased by FBS supplementation during the late IVC phase. In addition, treatment with FBS elevated antioxidant transcript levels during the late IVC phase. In contrast, supplementation with FBS during the entire period (1–6 days) or during the early IVC phase (1–2 days) greatly impaired the developmental parameters. Consistent with the results from PA embryos, the developmental competence of *in vitro* fertilization (IVF) or somatic cell nuclear transfer (SCNT) embryos were markedly improved by treatment with FBS during the late IVC phase. Moreover, the embryonic stage-specific effects of FBS were reversed by the addition of an oxidant and were mimicked by treatment with an antioxidant. These findings may increase our understanding of redox-dependent early embryogenesis and contribute to the large-scale production of high-quality IVP embryos.

## Introduction

Pigs are considered useful experimental animals for biomedical research, such as xenotransplantation and new-drug discovery, due to their high similarity to humans in anatomical and physiological features [1]. For this reason, *in vitro*-produced (IVP) porcine embryos have been used extensively to generate transgenic animals used as research materials, such as bio-organs and disease models [2–4]. However, unlike *in vivo* embryos, poor developmental competence is a major obstacle in the mass production of high-quality porcine IVP embryos. To address this issue, many researchers have attempted to improve the quality of porcine IVP embryos by adding numerous supplements to *in vitro* culture (IVC) medium to reduce the developmental damages caused by *in vitro* conditions [5–7]. However, IVC methods for porcine IVP embryos require further improvement by the application of new IVC supplements and by elucidation of the underlying mechanism(s).

Animal serum is a common supplement of IVC medium used for mammalian cell culture including early embryos, and it supplies important components, such as nutrients, pH buffers and anti-oxidants [8–10]. Addition of serum is often beneficial for IVC of mammalian oocytes and embryos [11], as evidenced by improved oocyte maturation, zona pellucida hardening prevention and increased blastocyst development rate [12–14]. In particular, fetal bovine serum (FBS) or fetal calf serum (FCS) is widely used due to several advantageous components, such as low levels of antibodies and numerous growth factors [15]. Blastocyst development and hatching rate are improved by replacing the medium with IVC medium containing FBS or FCS after late phase of IVC in pig, mouse and rat [16–19]. However, studies regarding the effects of serum supplementation on embryonic stage and the underlying mechanism(s) are lacking.

Reactive oxygen species (ROS) are generated as a byproduct of cellular metabolism [20, 21]. The primary role of ROS is highly associated with cellular damage, whereas recent reports have revealed that ROS participate in the modulation of signal pathways governing various cellular behaviors, such as proliferation, differentiation and apoptosis [22, 23]. As shown in various living organisms or cells, IVP embryos that develop early suffer from oxidative stress caused by IVC conditions, leading to a reduction in developmental competence or quality of developing early embryos [24]. In particular, decreases in total or trophectoderm (TE) cells in IVP blastocysts are major problems in pig development compared to other species of domestic animals [25], which results in delayed post-implantation development [26]. Proportion of TE cells in porcine IVP blastocysts, compared to *in vivo* blastocysts, are approximately 46% and 60%, respectively, [27, 28]. Thus, many researchers have attempted to reduce oxidative stress levels by adding numerous supplements with ROS scavenging activity, such as anthocyanin [29], L-carnitine [30], hypotaurine [31], vitamin C [32], β-mercaptoethanol [33, 34] and selenium [35]. Although the effects of FBS supplementation on early development of mammalian IVP embryos have been reported, to our knowledge, no evidence has been published regarding the relationship between FBS and oxidative stress during the early development of mammalian embryos.

In the present study, we showed that the effect of FBS on early embryogenesis varied according to the supplementation period or developmental stage. Addition of FBS during the late IVC phase was beneficial to the early development of porcine IVP embryos, whereas developmental competence was decreased significantly by the use of medium containing FBS during the early IVC phase. Interestingly, ROS generation was reduced significantly by FBS treatment regardless of the culture period or developmental stage. Importantly, the effect of antioxidants on the early development of porcine embryos was similar to that of FBS, which was mostly ablated by oxidant treatment. These findings increase our understanding of redox status governing early embryogenesis and may contribute to mass-scale production of high-quality IVP embryos.

## Materials and methods

### Ethics statement

All procedures and use of pigs were approved by the Korea Research Institute of Bioscience and Biotechnology (KRIBB) Institutional Animal Care and Use Committee (Approval No. KRIBB-AEC-16075).

### Chemicals

All chemicals and reagents were purchased from Sigma-Aldrich Chemical Co. (St. Louis, MO, USA) unless otherwise indicated.

### Oocyte collection and *in vitro* maturation (IVM)

Porcine ovaries were obtained from a local slaughterhouse and transported to the laboratory in 0.9% saline supplemented with 75 μg/mL potassium penicillin G and 50 μg/mL streptomycin sulfate and maintained at 25–30°C. Intact cumulus oocyte complexes (COCs) were aspirated from follicles (3–8 mm in diameter) using a disposable 10-mL syringe with an 18-gauge needle. Aspirated COCs with at least three layers of compact cumulus cells and homogeneous cytoplasm were washed three times in low-carbonate tyrode’s albumin lactate pyruvate-HEPES (TL-HEPES) medium [36] containing 1 mg/mL polyvinyl alcohol (PVA). Approximately 70 oocytes were matured in 500 μL of the IVM medium in a four-well multi-dish (Nunc, Roskilde, Denmark) at 38.5°C in 5% CO_2_ in air. Tissue culture medium 199 (TCM-199) was used during the 44-h maturation. During the first period of maturation, 0–22 h, the medium was supplemented with 10% porcine follicular fluid (PFF), 0.57 mM cysteine, 25 μM β-mercaptoethanol, 10 ng/mL epidermal growth factor (EGF), 10 IU/mL pregnant mare serum gonadotropin (PMSG) and 10 IU/mL human chorionic gonadotropin (hCG). During the second stage (22–44 h), the same media was used without hormones. After IVM, the COCs were exposed briefly to 0.1% hyaluronidase and gently aspirated using a small bore glass pipette to remove the cumulus cells. Only oocytes with a visible polar body, regular morphology and homogenous cytoplasm were used.

### Parthenogenetic activation (PA)

Matured oocytes were placed on a 1-mm gab wire chamber (CUY5000P1, Nepagene, Chiba, Japan) overlaid with 10 μL 280 mM mannitol solution containing 0.1 mM MgSO_4_∙7H_2_O, 0.1 mM CaCl_2_∙2H_2_O, 0.5 mM HEPES, 0.01% PVA, as described previously [37]. Immediately, oocytes were activated with 110 V DC for 50 μsec using an electro cell fusion generator (LF101, Nepagene). Approximately activated oocytes were transferred into the activation medium, IVC medium [porcine zygote medium-3 (PZM-3) medium containing 0.4% BSA] supplemented with 5 μg/mL cytochalasin B (CB) and 2 mM 6-dimethylaminopurine (6-DMAP) for 4 h at 38.5°C in 5% CO_2_ in air. After 4 h, the oocytes were washed in IVC medium and transferred to fresh IVC medium at 38.5°C in 5% CO_2_ in air. Cleavage and blastocyst formation were evaluated at days 2 and 6, respectively.

### *In vitro* fertilization (IVF)

*In vitro* fertilization (IVF) of porcine oocytes was performed as described previously [29]. IVF was performed in modified Tris-buffered medium (mTBM) consisting of 113.1 mM NaCl, 3 mM KCl, 7.5 mM CaCl_2_∙2H_2_O, 20 mM Tris (crystallized free base; Fisher Scientific, Waltham, MA, USA), 11 mM glucose and 5 mM sodium pyruvate. Ejaculated fresh semen from swine was washed three times by centrifugation (100 g for 3 min at room temperature) with Dulbecco’s phosphate-buffered saline (DPBS; Gibco-BRL, NY, USA) supplemented with 1 mg/mL BSA, 100 μg/mL penicillin G and 75 μg/mL streptomycin sulfate. After the washing procedure was complete, the spermatozoa were re-suspended in mTBM at pH 7.8. The oocytes were washed three times in mTBM with 2.5 mM caffeine sodium benzoate and 1 mg/mL BSA and placed into 48 μL mTBM under mineral oil. Next, 2 μL diluted spermatozoa was added to 48 μL mTBM containing 10–15 oocytes, yielding a final concentration of 1.5ⅹ10^5^ spermatozoa/mL. The oocytes were co-incubated with the spermatozoa for 6 h at 38.5°C in an atmosphere of 5% CO_2_ in air. After 6 h, the oocytes were stripped by gentle pipetting and transferred to IVC medium. The IVF embryos were washed three times in fresh IVC medium, transferred into 40 μL IVC droplets under mineral oil and then stored at 38.5°C and 5% CO_2_ in air for 6 days. Cleavage and blastocyst formation were evaluated at days 2 and 6, respectively.

### Preparation of donor cells

Donor cells were obtained from cultured minced porcine kidney tissue. The kidney was obtained from a neonatal pig (2 days old, male) by surgical operation. Harvest kidney tissues were stored in DPBS washing buffer containing 10% (v/v) penicillin/streptomycin (Pen/Strep; Invitrogen, Carlsbad, CA, USA) on ice until isolation. Small pieces of kidney tissue were placed in 60-mm culture dishes and cultured in Dulbecco’s Modified Eagle’s Medium (DMEM; Invitrogen, Carlsbad, CA, USA) containing 10% fetal bovine serum (FBS; 16000–044, GIBCO, Carlsbad, CA, USA), 10 ng/mL basic fibroblast growth factor (bFGF) and 1% penicillin/streptomycin (Invitrogen) at 38.5°C in 5% CO_2_ in air. These porcine kidney-derived fibroblasts were used at passages 4–6. The cells were synchronized at the G0-G1 phase of the cell cycle by an additional 3 days of culture, without changing the medium, after reaching confluency.

### Somatic cell nuclear transfer (SCNT)

After IVM, matured oocytes with a visible first polar body were selected for somatic cell nuclear transfer (SCNT). Cumulus-free oocytes in DPBS supplemented with 4 mg/mL BSA, 75 μg/mL penicillin G, 50 μg/mL streptomycin sulfate and containing 7.5 μg/mL CB were penetrated by cutting the pipette to make a slit and then squeezing approximately 10% of the cytoplasm and the first polar body out of the oocytes under an automated inverted microscope (DMI 6000B, Leica, Wetzlar, Germany) equipped with a micromanipulator (NT-88-V3, Nikon-Narishige, Tokyo, Japan). Porcine kidney cells that were round, 15–20 μm in diameter and had good refractivity were selected and placed into the perivitelline space through the near slit in the zona pellucida made during enucleation. The oocyte-cell couplets were maintained in IVC medium at 38.5°C in 5% CO_2_ in air until electrical fusion. A single cell-oocyte couplet was equilibrated in fusion medium consisting of 280 mM mannitol containing 0.1 mM MgSO_4_∙7H_2_O and 0.01% PVA [37], then sandwiched between two parallel electrodes (100-μm diameter; CUY 5100–100, Nepagene) attached to the micromanipulator, rotated to the contact surface between the cytoplasm and the donor cell perpendicular to the electrodes and activated by one direct current pulse of 23 V for 50 μsec using an electro cell fusion generator and incubated at 38.5°C in 5% CO_2_ in air. After 2 h, oocyte–cell couplets that were completely fused, as observed under an automated inverted microscope, were selected, then transferred to a 1-mm gab wire chamber overlaid with 10 μL 280 mM mannitol solution containing 0.1 mM MgSO_4_∙7H_2_O, 0.1 mM CaCl_2_∙2H_2_O, 0.5 mM HEPES and 0.01% PVA and activated with 110 V DC for 50 μsec using an electro cell fusion generator. The electro-activated oocytes were transferred into the chemically assisted activation medium supplemented with 50 nM trichostatin A (TSA) for 4 h at 38.5°C in 5% CO_2_ in air. After 4 h, activated embryos were transferred to IVC medium supplemented with 50 nM TSA and cultured for 20 h. After 20 h, SCNT embryos were washed in IVC medium and transferred to IVC medium at 38.5°C in 5% CO_2_ in air. Cleavage and blastocyst formation were evaluated at days 2 and 6, respectively.

### Chemical treatment

To investigate the effects of FBS (GIBCO) on early embryogenesis and the related signaling pathways, the presumed zygotes were cultured in IVC medium supplemented with 10% FBS in the presence or absence of glutathione (GSH; 0.5 and 1 mM), hydrogen peroxide (H_2_O_2_; 0.1 and 0.5 mM), SB203580 [p38 mitogen-activated protein kinase (MAPK) inhibitor; 10 μM] or LY294002 (AKT inhibitor; 10 μM) for various culture periods (absence, day 0; entire period, days 0–6 of IVC; early phase, days 0–1 or 0–2 of IVC; late phase, days 4–6 or 5–6 of IVC). All chemicals were provided as lyophilized powders and dissolved in porcine zygote culture medium or dimethyl sulfoxide. The respective diluents were used as negative controls in each experiment.

### Terminal deoxynucleotidyl transferase-mediated dUTP-digoxygenin nick end-labeling (TUNEL) assay

To detect apoptotic cells in the blastocysts, the terminal deoxynucleotidyl transferase-mediated dUTP-digoxygenin nick end-labeling (TUNEL) assay was conducted using an *In Situ* Cell Death Detection Kit (Roche, Basel, Swiss). Blastocysts on day 6 were washed three times with 0.1% PVA in (PBS-PVA) and fixed in 4% (v/v) paraformaldehyde diluted in PBS for 1 h at room temperature. For membrane permeabilization, the fixed embryos were incubated in PBS containing 1% (v/v) Triton X-100 for 1 h at room temperature. Subsequently, the embryos were washed three times in PBS-PVA and stained with fluorescein-conjugated dUTP and terminal deoxynucleotidyl transferase for 1 h at 38.5°C. As a positive control for the TUNEL reaction, a group of blastocysts was incubated in 1000 U/mL deoxyribonuclease at 38.5°C for 10 min, followed by TUNEL staining. Another group of blastocysts was incubated in fluorescein-dUTP in the absence of terminal deoxynucleotidyl transferase as a negative control. Subsequently, the blastocysts were washed three times in PBS-PVA and mounted on slides with a mounting solution containing 1.5 g/mL 4, 6-diamidino-2-phenylindole (DAPI; Vector Laboratories Inc., Burlingame, CA, USA). DAPI-labeled or TUNEL-positive nuclei were observed under a fluorescence microscope, and the number of apoptotic nuclei and total number of nuclei were counted. These experiments were performed in triplicate with 10–15 blastocysts per group for each experiment.

### Differential staining

Blastocysts were fixed in 4% paraformaldehyde for 40 min and washed three times in PBS-PVA for 10 min each. For membrane permeabilization, the fixed blastocysts were incubated in PBS containing 0.2% (v/v) Triton X-100 for 40 min at room temperature. Subsequently, blastocysts were washed three times in PBS-PVA and stored in PBS-PVA supplemented with 1% BSA (PBS-PVA-BSA) at 4°C overnight. The blastocysts were blocked with 10% normal goat serum (NGS) for 45 min and then incubated overnight at 4°C with primary antibody, mouse monoclonal anti-Cdx2 (an undiluted solution; Biogenex Laboratories Inc., San Ramon, CA, USA). Subsequently, the blastocysts were washed three times in PBS-PVA-BSA for 10 min each and incubated for 40 min at room temperature with conjugated secondary antibodies, Alexa-Fluor-488-labeled goat anti-mouse IgG (1:200 in PBS-PVA-BSA). After the blastocysts were washed three times in PBS-PVA-BSA for 10 min each, the DNA was stained with 2 μg/mL DAPI. DAPI-labeled or Cdx2-positive nuclei were observed using a fluorescence microscope (Olympus, Tokyo, Japan).

### Measurement of intracellular ROS

Measurement of ROS levels in embryos was performed as described previously [38]. 5-(and-6)-chloromethyl-2ʹ,7ʹ-dichlorodihydro-fluorescein diacetate, acetyl ester (CM-H_2_DCFDA; Invitrogen) was used to detect intracellular ROS as green fluorescence. At least 10 embryos from each treatment group were incubated for 20 min in a solution of TL-HEPES-PVA mixed with 5 μM CM-H_2_DCFDA. After incubation, embryos were washed with DPBS containing 0.1% (w/v) PVA and aliquoted into 10 μL droplets; fluorescence was observed under a fluorescence microscope (DMI 4000B; Leica) with ultraviolet filters (460 nm for ROS). Fluorescent images were saved as graphic files in tagged image file format. The fluorescence intensities of the embryos were analyzed using Image J software (version 1.47; National Institute of Health, Bethesda, MD, USA) and normalized to those of control embryos.

### Quantitative real-time polymerase chain reaction (qRT-PCR)

Poly(A) mRNAs were extracted from 10 blastocysts using the Dynabeads mRNA Direct kit (Invitrogen Dynal AS, Oslo, Norway) according to the manufacturer’s protocol. Briefly, after thawing, samples were lysed in 300 μL lysis/binding buffer at room temperature for 10 min, and 10 μL Dynabeads oligo(dT)_25_ were added to each sample. The beads were hybridized for 5 min and then separated from the binding buffer using a Dynal magnetic bar (Invitrogen). Bound poly(A) mRNAs and beads were washed with buffers A and B and then separated by adding 10 μL Tris buffer. The resulting poly(A) mRNAs were reverse-transcribed in 20 μL reactions containing oligo(dT)_20_, 5ⅹRT buffer (containing 25 mM Mg^2+^), 10 U of the RNase inhibitor ReverTra Ace (Toyobo, Osaka, Japan) and a 10 mM mixture of dNTPs. Secondary RNA structure was denatured by incubating at 42°C for 60 min to facilitate cDNA production. The reaction was terminated by incubation at 99°C for 5 min. The resulting cDNA was used as a template for PCR amplification. The following PCR conditions were used: 95°C for 30 s, 60°C for 30 s and 72°C for 30 s, followed by extension at 72°C for 5 min. The Mx3000P QPCR system (Agilent, Santa Clara, CA, USA) and SYBR premix Ex Taq (Takara Bio Inc., Shiga, Japan) were used for quantitative real-time polymerase chain reaction (qRT-PCR). The threshold cycle (Ct) is defined as the fractional cycle number at which the fluorescence passes a fixed threshold above baseline. For the comparative analyses, mRNA expression levels were normalized to glyceraldehyde-3-phosphate dehydrogenase (GAPDH) and are expressed as the fold change. The sample delta Ct (^SΔCT^) value was calculated from the difference between the Ct values of GAPDH and the target genes. The relative gene expression levels between the samples and the controls were determined using the formula 2^−^(^SΔCT−CΔCT^). The primers used in the current study are listed in S1 Table.

### Western blot analysis

Twenty embryos in each group were washed twice with PBS and lysed in 20 μl lysis buffer (20 mM HEPES, 150 mM NaCl, 2 mM EGTA, 1 mM EDTA, 20 mM glycerol phosphate, 1% Triton X-100 and 10% glycerol) containing protease inhibitor cocktail for 1 h subsequently boiled at 100°C for 5 min. The protein samples were separated on a 10% SDS-polyacrylamide gel electrophoresis gel (iNtRON Biotechnology, Gyeonggi-do, Korea) and transferred to a Polyvinylidene difluoride (PVDF) membranes (Millipore, Billerica, MA, Canada). The membrane was blocked in Tris-buffered saline (TBS) containing 0.25% Tween 20 (TBST) and 5% BSA for 1 h, rinsed in TBST, and probed with an anti-phospho-p38-mitogen-activated protein kinase (MAPK) [phospho-p38 (Thr180/Tyr182), 1:1000 dilution; Cell Signaling Technology, Beverly, MA, USA], anti-p38-MAPK (1:1000 dilution; Cell Signaling Technology), anti-phospho-AKT [p-AKT (ser473) 1:1000 dilution; Cell Signaling Technology] and AKT (1:1000 dilution; Cell Signaling Technology), anti- antibodies at 4°C overnight. The blot was washed with TBST and subsequently incubated with horseradish peroxidase-conjugated secondary antibody. The membranes were visualized using enhanced chemiluminescence detection reagent (Thermo Fisher scientific, Walltham, MA, USA) according to the manufacturer’s instructions.

### Statistical analyses

All experiments were repeated at least three times, and data are presented as the means ± standard error (SE). Differences in the mean percentages of blastocysts and hatching blastocysts among the treatments were compared using factorial ANOVA, followed by Duncan’s multiple range tests using sigma stat software (Systat Software Inc., San Jose, CA, USA). *P-*values less than 0.05 were considered statistically significant.

## Results

### Supplementation with FBS during late IVC improves developmental competence in porcine PA embryos

**Fig 1:**
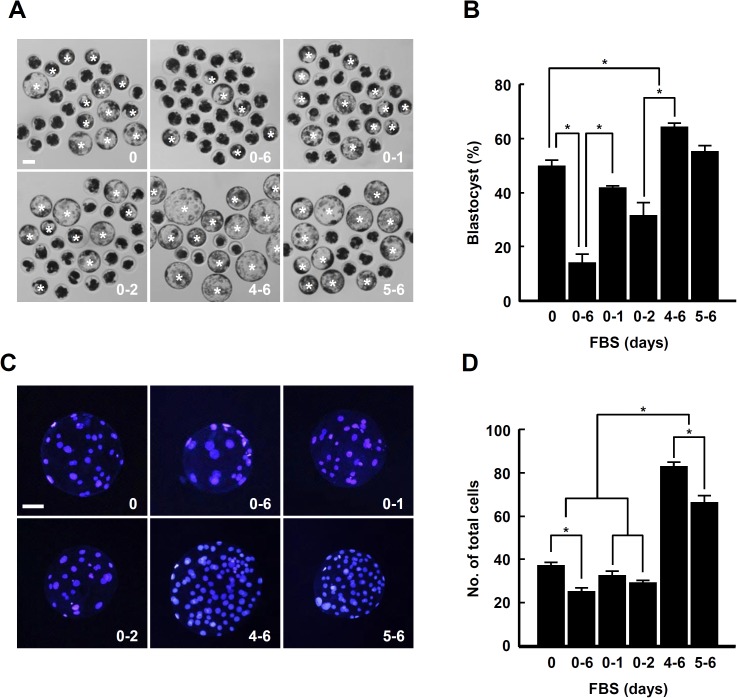
Effect of FBS supplementation timing on the developmental competence of porcine PA embryos. (A) Representative photographs of blastocysts (white asterisks) developed in the presence or absence of FBS during the entire culture period (0–6 days), early phase (0–1 and 0–2 days) and late phase (4–6 and 5–6 days) of IVC. Bar = 50 μm. (B) Quantification of the blastocyst developmental rate in the indicated groups. The data are from three independent experiments, and values represent the means ± SE (^*^P < 0.05). (C and D) DAPI staining for scoring the number of cells in blastocysts of the indicated groups (C) and quantification of the total cell number (D). Bar = 50 μm (C). The data are from three independent experiments, and values represent the means ± SE (^*^P < 0.05; D).

To study the effect of FBS on the early development of porcine embryos, PA embryos were cultured in IVC medium supplemented with 10% FBS for various culture periods (absence, day 0; entire period, days 0–6 of IVC; early phase, days 0–1 or 0–2 of IVC; late phase, days 4–6 or 5–6 of IVC), and the blastocyst development rate and total cell number were assessed after 6 days of IVC. In particular, blastocyst development rate and total cell number were the lowest in the entire culture period treatment group, whereas both parameters were most notably increased in the 4–6 day treatment group (Fig 1 and S2 Table). Consistent with these results, the proportion of hatched or hatching blastocysts was markedly increased by treatment with FBS during the late phase, especially for 4–6 days, compared to the other groups (S1 Fig and S3 Table).

### The effect of ROS levels in early phase treatment of FBS during early embryogenesis of porcine PA embryos

**Fig 2:**
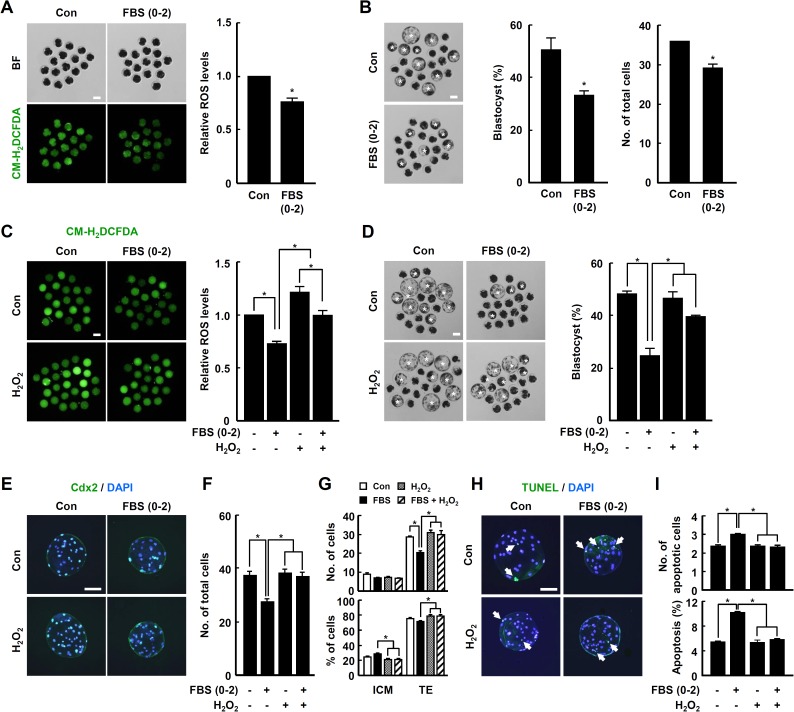
Involvement of ROS in FBS-associated phenotypes during early IVC phase of PA embryos. (A) Fluorescence microscopy of 4- to 8-cell embryos treated with CM-H_2_DCFDA after treatment with FBS for 48 h (left panel) and quantification of ROS levels (right panel) in the indicated groups. Bar = 100 μm. (B) Representative photographs of the blastocysts (left panel; white asterisks) and quantification of the blastocyst developmental rate (center panel) and total cell number (right panel) within the indicated groups. Bar = 100 μm. (C) Fluorescence microscopy of 1-cell embryos treated with CM-H_2_DCFDA after 6 h of cultivation in the presence or absence of either FBS and/or 0.5 mM hydrogen peroxide (left panel) and quantification of ROS levels (right panel) within the indicated groups. Bar = 100 μm. (D) Representative photographs of the developed blastocysts (left panel; white asterisks) and quantification of the blastocyst developmental rate within the indicated groups. Bar = 100 μm. (E–G) Immunocytochemical analysis of Cdx2/DAPI using blastocysts developed under the indicated IVC conditions for 0–2 days of IVC (E) and quantification of the total cell number (F) and ICM/TE proportions (G) in the indicated groups. Merged images (light green) between Cdx2 (green) and DAPI (blue) signals are shown (E). Bar = 100 μm (E). The data are from three independent experiments, and values represent the means ± SE (^*^P < 0.05; F and G). (H and I) Apoptosis detection analysis using blastocysts developed under the indicated IVC conditions for 0–2 days (H), and quantification of the number and proportion of apoptotic cells in the indicated groups (I). Merged images (light green) between TUNEL (green, white arrow) and DAPI (blue) signals are shown (H). Bar = 50 μm (H). The data are from three independent experiments, and values represent the means ± SE (^*^P < 0.05; I).

To investigate ROS generation during whole embryo developmental period, PA embryos were cultured in IVC medium. ROS levels were continuously increased during early embryogenesis, whereas the gradual decreases in antioxidant enzyme transcripts, including *SOD1*, *GPx1*, *Prdx2*, and *Catalase*, were found (S2 Fig), indicating possible role of the antioxidant enzymes as embryonic ROS scavengers. To investigate the relationship between FBS supplementation and ROS levels during early stage embryo development, PA embryos were cultured in the presence or absence of FBS for 0–2 days of IVC, and ROS reduction was determined using CM-H_2_DCFDA and subjected to fluorescence microscopy (Fig 2A). As observed in the FBS treatment group, the blastocyst developmental rate and total cell number were significantly decreased relative to the control group (Fig 2B and S4 Table). Consistent with this, the embryonic ROS level was successfully decreased by adding 1 mM GSH, which led to significant decreases in cleavage rate, blastocyst development rate, TE cell number, and cellular survival compared with controls (S3 Fig, S5 and S6 Tables). However, little or no changes concerning ROS levels and blastocyst development rate were found following treatment with low GSH concentrations, such as 0.1, 0.2 and 0.5 mM (data not shown). In contrast, the decreases in cleavage and blastocyst developmental rates were significantly restored by the addition of hydrogen peroxide (S4 Fig and S7 Table). Consistent with, elevation of ROS levels by the addition of hydrogen peroxide in the FBS treatment group restored the blastocyst formation and proportion of TE cells (Fig 2C–2G, S8 and S9 Tables). Similarly, TUNEL analysis of blastocyst showed that the proportion of apoptotic cells significantly decreased in the presence of FBS and hydrogen peroxide to compare the control and only FBS groups (Fig 2H and 2I and S9 Table).

### The effect of ROS levels in late phase treatment of FBS during early embryogenesis of porcine PA embryos

**Fig 3:**
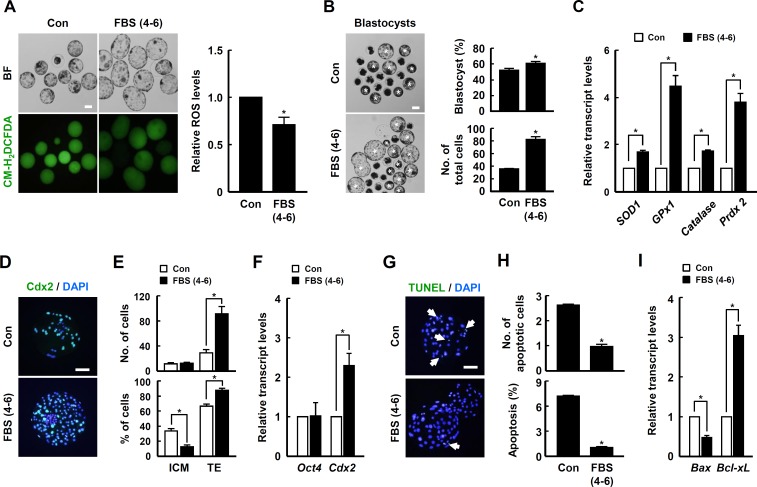
Effects of FBS supplementation during late IVC phase on the developmental competence of PA embryos. (A) Bright field (top of left panel) and fluorescence (bottom of left panel) images of blastocysts treated with CM-H_2_DCFDA in the indicated groups (left panel) and quantification of ROS levels in the indicated groups (right panel). Bar = 100 μm. The data are from three independent experiments, and values represent the means ± SE (^*^P < 0.05). (B) Representative photographs of the developed blastocysts (white asterisks) and quantification of the blastocyst development rate (top of right panel) and total cell number (bottom of right panel) in the indicated groups. Bar = 100 μm. The data are from three independent experiments, and values represent the means ± SE (^*^P < 0.05). (C) qRT-PCR analysis of the relative abundances of antioxidant genes *SOD1*, *GPx1*, *Catalase* and *Prdx2* in blastocysts from the indicated groups. The data are from three independent experiments, and values represent the means ± SE (^*^P < 0.05). (D) Immunocytochemical analysis of Cdx2 using blastocysts cultured in the presence or absence of FBS for 4–6 days of IVC. Merged images (light green) between Cdx2 (green) and DAPI (blue) signals are shown. Bar = 50 μm. (E) Quantification of the total cell number (top panel) and TE/ICM proportions (bottom panel) in the indicated groups. The data are from three independent experiments, and values represent the means ± SE (^*^P < 0.05). (F) qRT-PCR analysis of the relative abundances of *Oct4* and *Cdx2* transcript levels in blastocysts from the indicated groups. The data are from three independent experiments, and values represent the means ± SE (^*^P < 0.05). (G) Apoptosis detection analysis in blastocysts from the indicated groups. Merged images (light green) between TUNEL (green, white arrow) and DAPI (blue) signals are shown. Bar = 50 μm. (H) Quantification of the number (top panel) and proportion (bottom panel) of apoptotic cells in the indicated groups. The data are from three independent experiments, and values represent the means ± SE (^*^P < 0.05). (I) qRT-PCR analysis of the relative abundance of *Bax* and *Bcl-xL* transcript levels in blastocysts from the indicated groups. The data are from three independent experiments, and values represent the means ± SE (^*^P < 0.05).

To investigate the effect of FBS on the late phase development of porcine embryos, PA embryos were cultured in IVC medium supplemented with 10% FBS for 4–6 day. In particular, blastocyst development rate and total cell number were most notably increased in the 4–6 day FBS treatment group (Fig 3A and 3B and S10 Table) Based on qRT-PCR analysis, the transcript levels of antioxidant enzymes, including *SOD1*, *GPx1*, *Catalase* and *Prdx2*, were significantly upregulated by the addition of FBS during the late IVC phase compared to control (Fig 3C).

To clarify the beneficial role of FBS during the late IVC phase of early embryogenesis, porcine PA embryos were cultured in the presence or absence of 10% FBS for 4–6 days of IVC, and the ICM/TE proportion and apoptotic cell number were scored based on immunocytochemical analyses of Cdx2 and TUNEL, respectively, using blastocysts from each group. Compared to control, the ICM proportion was decreased markedly in the FBS treatment group, whereas the TE proportion was increased markedly (Fig 3D and 3E and S11 Table). Similarly, the *Cdx2* transcript level was significantly upregulated with FBS treatment compared to control (Fig 3F). Moreover, the number and proportion of apoptotic cells were notably decreased in the FBS treatment group (Fig 3G and 3H and S11 Table). Similarly, reduction of the pro-apoptotic *Bax* transcript level and elevation of the anti-apoptotic *Bcl-xL* transcript level were detected in the FBS treatment group compared to control (Fig 3I).

**Fig 4:**
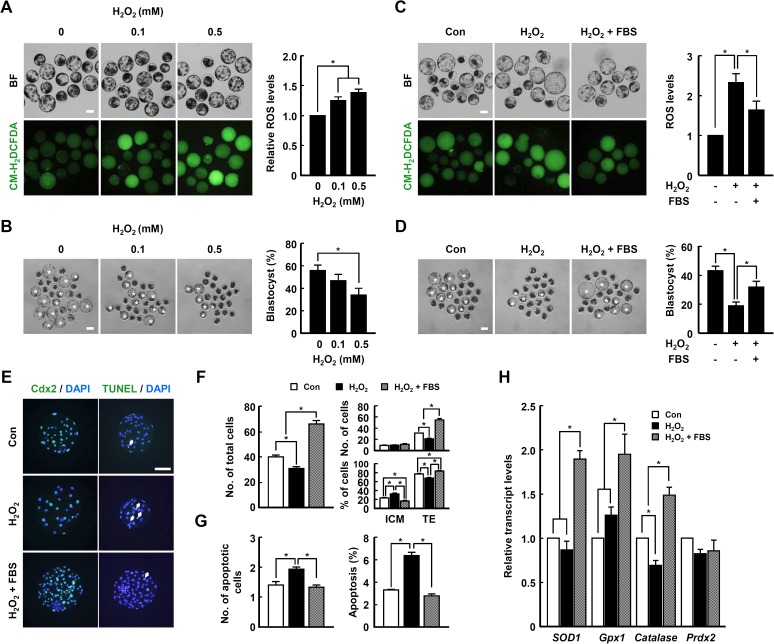
Involvement of ROS in FBS-associated phenotypes during late IVC phase of PA embryos. (A) Fluorescence microscopy of blastocyst treated with CM-H_2_DCFDA after cultivation in the presence or absence of hydrogen peroxide (0.1 and 0.5 mM) for 4–6 days of IVC (bottom of left panel) and bright field (BF; top of left panel) and quantification of ROS generation (right panel) within the indicated groups. (B) Representative photographs of the developed blastocysts (white asterisks; left panel) and quantification of blastocyst developmental rate (right panel) within the indicated groups. Bar = 100 μm. The data are from three independent experiments, and values represent the means ± SE (^*^P < 0.05). (C) Fluorescence microscopy of blastocysts treated with CM-H_2_DCFDA after cultivation in the presence or absence of FBS and/or 0.5 mM hydrogen peroxide for 4–6 days of IVC (top of left panel) and bright field (BF; top of left panel) and quantification of ROS generation (right panel) within the indicated groups. (D) Representative photographs of the developed blastocysts (white asterisks; left panel) and quantification of blastocyst rate (right panel). The data are from three independent experiments, and values represent the means ± SE (^*^P < 0.05). (E-G) Immunocytochemical analysis of Cdx2 (left panel) and TUNEL staining (right panel). Bar = 50 μm. Quantification of the total cell number (F; left panel) ICM/TE proportion (F; right panel) and proportion of apoptotic cells (G) and in the indicated groups. The data are from three independent experiments, and values represent the means ± SE (^*^P < 0.05). (H) qRT-PCR analysis of the relative abundance of antioxidant genes *SOD1*, *GPx1*, *Catalase* and *Prdx2* in blastocysts from the indicated groups. The data are from three independent experiments, and values represent the means ± SE (^*^P < 0.05).

To examine the effect of oxidant treatment during the late IVC phase on early embryogenesis. PA embryos were cultured in the presence or absence of FBS or hydrogen peroxide for 4–6 days. Unlike the early IVC phase, elevation of ROS levels due to the addition of hydrogen peroxide for 4–6 days of IVC significantly decreased the blastocyst development rate in a dose-dependent manner (Fig 4A and 4B and S12 Table). In addition, the detrimental effects of hydrogen peroxide treatment for 4–6 days of IVC on blastocyst development rate, TE proportion and cellular survival were greatly ameliorated by co-treatment with FBS (Fig 4C–4G, S13 and S14 Tables). Interestingly, the transcript levels of antioxidant enzymes, including *SOD1*, *GPx1* and *Catalase*, were upregulated by FBS treatment (Fig 4H).

### Beneficial role of FBS in late IVC phase involves p38 MAPK and AKT cascades

**Fig 5:**
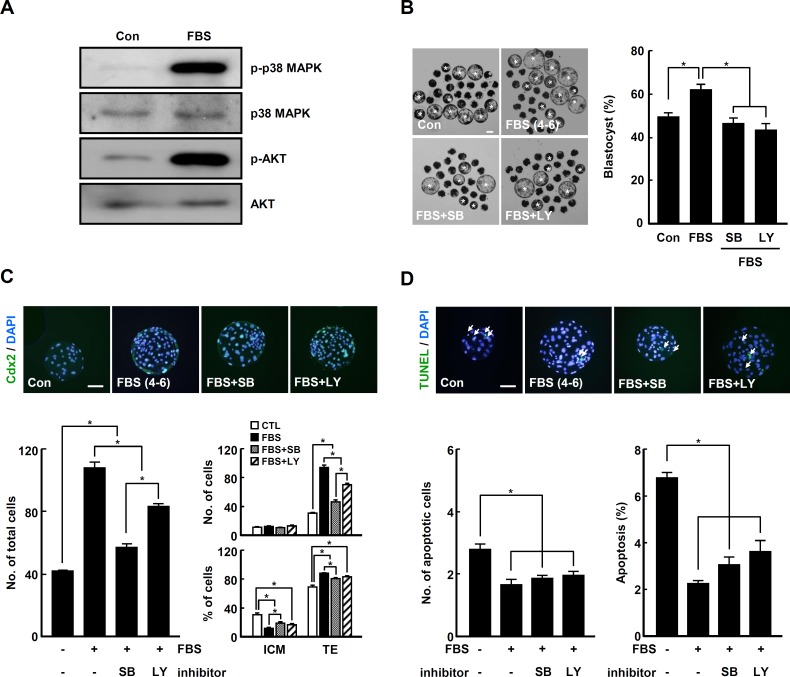
Involvement of p-p38 MAPK and p-AKT cascades in FBS-associated phenotypes during late IVC phase. (A) Western blot analysis of p-p38 MAPK and p-AKT in control and FBS supplementation during late phase in porcine PA embryos. (B) Representative photographs of the developed blastocysts (white asterisks; left panel) and quantification of blastocyst developmental rate (right panel) in the indicated groups. Bar = 100 μm. The data are from three independent experiments, and values represent the means ± SE (*P < 0.05). (C) Immunocytochemical analysis of Cdx2/DAPI. Merged images (light green) between Cdx2 (green) and DAPI (blue) signals are shown (top panel) and quantification of the total cell number (bottom left panel) and ICM/TE proportion (bottom right panel) in the indicated groups. Bar = 50 μm. (D) Apoptosis detection analysis in blastocysts from the indicated groups. Merged images (light green) between TUNEL (green, white arrow) and DAPI (blue) signals are shown (top panel) and quantification of apoptotic cells (bottom panel). Bar = 50 μm. The data are from three independent experiments, and values represent the means ± SE (^*^P < 0.05).

Based on the previous evidences concerning the close association of developmental competence with activation of p38 MAPK and AKT signaling pathways [39, 40], we investigated whether FBS supplementation in late IVC phase modulated p-p38 MAPK and p-AKT levels in PA blastocysts. Compared to control, the levels of p-p38 MAPK and p-AKT in PA blastocysts were markedly increased in FBS treatment group (Fig 5A). Interestingly, addition of either p38 MAPK inhibitor SB203580 (10 μM) or AKT inhibitor LY294002 (10 μM) significantly decreased the blastocyst development rate and TE proportion (Fig 5B and 5C, S15 and S16 Tables). However, no changes in proportion of apoptotic cells were found in the inhibitor treatment groups compared to FBS treatment group (Fig 5D and S16 Table).

### Treatment with FBS during the late IVC phase supports early embryogenesis of IVF and SCNT embryos

**Fig 6:**
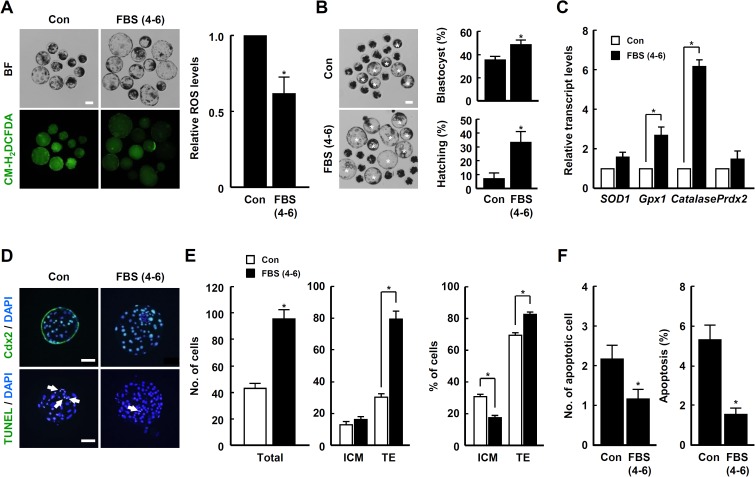
Beneficial effect of FBS during late IVC phase on developmental competence of porcine IVF embryos. (A) Bright field (top panel) and fluorescence (bottom panel) images of blastocysts treated with CM-H_2_DCFDA after cultivation in the presence or absence of FBS for 4–6 days of IVC (left panel) and quantification of ROS levels in the indicated groups (right panel). Bar = 100 μm. The data are from three independent experiments, and values represent the means ± SE (^*^P < 0.05). (B) Representative photographs of the blastocysts (white asterisks; left panel) developed in the presence or absence of FBS for 4–6 days of IVC after IVF and quantification of blastocyst developmental (center panel) and hatching (right panel) rates in the indicated groups. The data are from three independent experiments, and values represent the means ± SE (^*^P < 0.05). (C) qRT-PCR analysis of the relative abundances of antioxidant genes *SOD1*, *GPx1*, *Catalase* and *Prdx2* in blastocysts from the indicated groups. The data are from three independent experiments, and values represent the means ± SE (^*^P < 0.05). (D) Immunocytochemistry of Cdx2 (top panel) and TUNEL staining (bottom panel) in blastocysts from the indicated groups. Bar = 50 μm. Merged images (light green color) between green (Cdx2, top panel; TUNEL, bottom panel) and blue (DAPI; both panels) signals are shown. (E and F) Quantification of ICM/TE (E) and apoptotic cell (F) number and proportion in the indicated groups. The data are from three independent experiments, and values represent the means ± SE (^*^P < 0.05).

Next, we investigated whether FBS supplementation for 4–6 days of IVC continues to benefit early embryogenesis of IVF and SCNT embryos. ROS levels were greatly reduced by FBS treatment (Fig 6A), leading to improved blastocyst development and hatching rates (Fig 6B and S17 Table). In addition, the transcript levels of antioxidant enzymes, including *SOD1*, *GPx1* and *Catalase*, were relatively high in the FBS treatment group compared to control (Fig 6C). Consistent with these results, the proportion of TE cells and cellular survival were significantly increased with FBS treatment (Fig 6D–6F and S18 Table).

**Fig 7:**
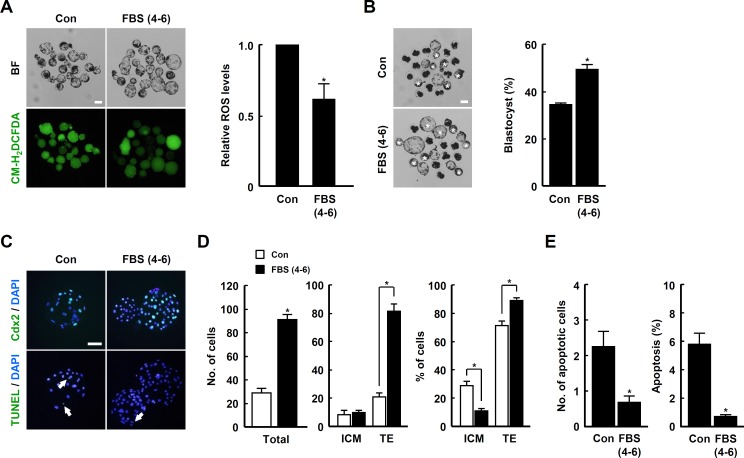
Beneficial effect of FBS during late IVC phase on developmental competence of porcine SCNT embryos. (A) Bright field (top of left panel) and fluorescence (bottom of left panel) images of SCNT blastocysts treated with CM-H_2_DCFDA after cultivation in the presence or absence of FBS for 4–6 days of IVC (left panel) and quantification of ROS levels in the indicated groups (right panel). Bar = 100 μm. The data are from three independent experiments, and values represent the means ± SE (^*^P < 0.05). (B) Representative photographs of the blastocysts (white asterisks) developed in the presence or absence of FBS for 4–6 days of IVC after SCNT (left panel) and quantification of the blastocyst developmental rate in the indicated groups (right panel). The data are from three independent experiments, and values represent the means ± SE (^*^P < 0.05). (C) Immunocytochemical analysis of Cdx2 (top panel) and TUNEL staining (bottom panel) in SCNT blastocysts from the indicated groups. Merged images (light green color) between green (Cdx2, top panel; TUNEL, bottom panel) and blue (DAPI; both panels) signals are shown. Bar = 50 μm. (D and E) Quantification of ICM/TE (D) and apoptotic cell (E) number and proportion in the indicated groups. The data are from three independent experiments, and values represent the means ± SE (^*^P < 0.05).

To further examine the effect of FBS supplementation during the late phase of early embryogenesis, SCNT embryos were cultured in the presence or absence of FBS for 4–6 days of IVC. In agreement with other results of this study, FBS treatment led to reduced ROS levels (Fig 7A) as well as an increased rate of blastocyst development (Fig 7B and S19 Table). TE cell proportion and cellular survival in SCNT blastocysts were also improved by the addition of FBS during the late IVC phase (Fig 7C–7E and S20 Table).

## Discussion

Cultivation of mammalian embryos under *in vitro* conditions causes oxidative stress-associated damages [41], which aggravates developmental parameters such as blastocyst formation rate, TE cell number and cellular survival [28, 42]. Despite the improvement in developmental parameters by the addition of numerous supplements, such as cytokines [43, 44], growth factors [45–47] and hormones [48], the quality of IVP embryos, particularly in pigs, requires further improvement by the application of various supplements and by the elucidation of the underlying mechanism(s). In the present study, we determined the optimal FBS treatment conditions for successful early embryogenesis in pigs and the underlying mechanism in relation to oxidative stress. To our knowledge, this is the first study demonstrating that FBS contributes to reduced ROS levels governing early development of porcine IVP embryos.

FBS is frequently used as a supplement because it supplies a cocktail of essential factors for cellular behaviors, such as cell growth, proliferation and attachment [49]. As shown in the present study, several reports have revealed that addition of FBS during the late phase of preimplantation development is beneficial to the developmental competence of IVP early embryos [50–52]. However, studies regarding the molecular mechanism(s) governed by supplementation with FBS are limited. Thus, our findings concerning the relationship between FBS and ROS levels could provide important information for understanding early developmental events as well as the significance of ROS regulation under *in vitro* conditions. Contrary to the results obtained in the current study, the addition of FBS to IVC medium often triggers generation of ROS in mammalian cells [11, 19, 53, 54]. NADPH oxidases, representative ROS-generating enzymes, are activated in response to FBS supplementation, which leads to accelerated cell cycle progression [55]. In contrast, we found that numerous antioxidant transcripts, such as *SOD1*, *GPx1* and *Catalase*, were markedly upregulated by FBS treatment (Figs 3C and 6C). Together with the reverse correlation between ROS and antioxidant levels during early embryogenesis (S1 Fig), the findings can aid in understanding the involvement of antioxidants in embryonic redox homeostasis, although the exact role of the increased antioxidant levels was not addressed in the present study. We show that similar results were obtained irrespective of the lot and different batches of FBS (data not shown).

In general, ROS function as negative regulators of cell behavior by causing oxidative damage [56]. Excessive or cumulative ROS levels can damage various cellular components including nucleotides, proteins and membranes [57–59]. As shown in various mammalian cells, early development of domestic animal IVP embryos can be easily damaged by the addition of extrinsic oxidants, including hydrogen peroxide, due to elevated oxidative damage [41]. Apoptosis incidence and TE cell number appear to be closely associated with oxidative stress during the early development of embryos [60, 61]. Consistent with these demonstrations, we observed that reduction of ROS levels by treatment with FBS in late IVC phase of early embryogenesis led to significant increase in the rates of blastocyst development and hatching, TE proportion and cellular survival in IVP embryos (Figs 3, 6 and 7). Like as shown in murine species [39, 40], numerous studies have demonstrated that *in vitro* developmental competence is highly dependent on the quantity of TE cells in domestic animals, such as cattle and pigs [28, 62, 63]. Indeed, the parameters dictating *in vitro* developmental competence are frequently in good accordance with TE cell quantity in IVP blastocysts in domestic animals [64, 65]. Moreover, recent reports have shown that the number and proportion of TE cells, unlike ICM, are markedly reduced in IVP porcine blastocysts compared to *in vivo* blastocysts [66, 67]. Consistent with these demonstrations, the current study showed that modulation of ROS led to changes in the number of TE cells, but not ICM (Figs 2–4, 6 and 7; S3 Fig; S6, S9, S11, S14, S18, and S20 Tables). Additionally, the statistical significance of ICM proportion is merely caused by modulation of TE cell number (Figs 2–4, 6 and 7; S3 Fig; S6, S9, S11, S14, S18, and S20 Tables). These results suggest that embryonic ROS is closely associated with proliferation or differentiation of TE cells rather than ICM, and that the quantity of TE cells is a more suitable parameter for qualifying IVP porcine embryos. Moreover, we demonstrated for the first time that the improvement in early developmental competence of IVP embryos highly depended on the activation of p-p38 MAPK and p-AKT signal pathways (Fig 5).

In contrast to the negative role of ROS in cell regulation, numerous reports have shown that normal cellular events, such as proliferation and differentiation, require ROS generation. Embryonic stem cells require increased levels of ROS before commitment to cardiomyocytes and neurons [68–70]. In addition, ROS play a pivotal role in cell proliferation via regulation of cell cycle-associated machineries, such as cyclin and cyclin-dependent kinases. In agreement with these results, our findings support opposite effects of ROS on cell regulation, as evidenced by both the beneficial role of ROS during the early phase of preimplantation development (Figs 1 and 2) and detrimental effect of ROS during late phase development (Figs 1, 3 and 4). Thus, we proposed that porcine IVP early embryos may be a useful model for understanding the dual role of ROS in living organisms. Presently, we are further investigating the detailed molecular mechanism(s) or signaling pathways governed by embryonic stage-specific ROS.

**Fig 8:**
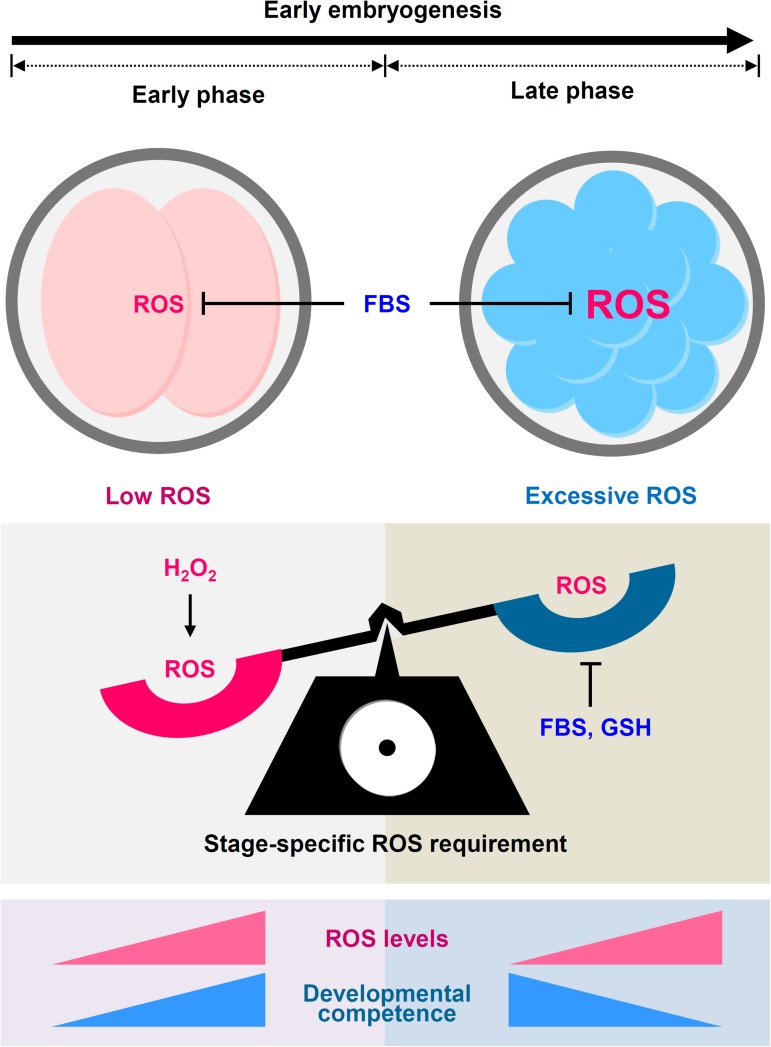
Hypothetical model for the differential effects of FBS on early embryogenesis. During IVC of porcine IVP embryos, FBS supplementation greatly reduced intracellular ROS levels regardless of the embryonic stage. Importantly, decreased ROS levels caused by FBS during the early IVC phase impaired early development of porcine IVP embryos, whereas FBS-mediated ROS reduction during the late IVC phase improved developmental competence. Similarly, addition of hydrogen peroxide and GSH during the early and late IVC phases, respectively, improved developmental competence. In conclusion, ROS requirement varied according to embryonic stage and was particularly greater during the early than during the late IVC phase, which can be modulated efficiently by FBS supplementation.

We have demonstrated that early development of porcine IVP embryos is highly dependent on the combination of FBS supplementation timing and embryonic ROS requirements (Fig 8). Reduced ROS levels during the early phase of preimplantation development impaired the developmental competence of porcine IVP embryos, which was completely reversed by decreased ROS levels during the late IVC phase. Thus, FBS-mediated ROS surveillance was beneficial only during the late phase, but not full-term or early IVC phase. Taken together, these results suggest that FBS can be used efficiently as a supplement in IVC for mass-scale production of high-quality porcine IVP embryos.

## Supporting information

S1 Fig Effect of FBS supplementation timing on the development of porcine PA blastocysts. (A and B) Representative photographs (A) and proportion (B) of the blastocysts at four different embryonic stages (EB, early blastocyst; MB, mid-blastocyst; ExB, expanded blastocyst; HB, hatched or hatching blastocyst).
(PDF)

S2 Fig ROS generation and antioxidant gene expression during early development of porcine PA embryos. (A) Fluorescence microscopy of 1-cell embryos to blastocysts treated with CM-H_2_DCFDA (left panel) and quantification of ROS levels (right panel) in the indicated groups. Bar = 100 μm. (B) qRT-PCR analysis of the relative abundance of *Sod1*, *Prx II*, *Gpx1* and *Bcl-xL* transcript levels in each developmental stages. The data are from three independent experiments, and values represent the means ± SE (^*^P < 0.05). Abbreviations are 1C, 1-cell; 2C, 2-cell; 4C, 4-cell; 6C, 6-cell; M, morula; BL, blastocyst.
(PDF)

S3 Fig Effects of FBS or antioxidant supplementation during the early IVC phase on ROS generation and developmental competence of porcine PA embryos. (A) Fluorescence microscopy of 1-cell embryos treated with CM-H_2_DCFDA after 6 h of cultivation in the presence or absence of either FBS or GSH (0.5 and 1.0 μM) for 0–2 days of IVC (left panel) and quantification of ROS level (right panel) The data are from three independent experiments, and values represent the means ± SE (^*^P < 0.05). (B) Representative photographs of the developed blastocysts (white asterisks; left panel) within the indicated groups and quantification of blastocyst developmental rate (right panel) in the indicated groups. Bar = 100 μm. The data are from three independent experiments, and values represent the means ± SE (^*^P < 0.05). (C) Immunocytochemical analysis of Cdx2 using blastocysts developed under the indicated IVC conditions for 0–2 days of IVC and quantification of the total cell number (C; bottom left panel) and ICM/TE proportions (C; bottom right panel) in the indicated groups. Merged images (light green) between Cdx2 (green) and DAPI (blue) signals are shown (C; top panel). Bar = 100 μm (C). The data are from three independent experiments, and values represent the means ± SE (^*^P < 0.05; C). (D) Apoptosis detection analysis using blastocysts developed under the indicated IVC conditions for 0–2 days (D), and quantification of the number and proportion of apoptotic cells in the indicated groups (D; bottom panel). Merged images (light green) between TUNEL (green, white arrow) and DAPI (blue) signals are shown (D; top panel). Bar = 50 μm (D). The data are from three independent experiments, and values represent the means ± SE (^*^P < 0.05; D).
(PDF)

S4 Fig Effect of ROS modulation during the early IVC phase on early development of porcine PA embryos. (A and B) Representative photographs of blastocysts (white asterisks; A) developed in the presence or absence of 1.0 μM GSH and/or 0.1 mM hydrogen peroxide for 0–2 days of IVC and quantification of cleavage and blastocyst developmental rates in the indicated groups (B). Bar = 50 μm. (A). The data are from three independent experiments, and values represent the means ± SE (^*^P < 0.05; B).
(PDF)

S1 Table Primer sequences used for semi-qRT-PCR and qRT-PCR. (PDF)

S2 Table Effect of FBS supplementation timing on the developmental competence of porcine PA embryos (PDF)

S3 Table Effect of FBS treatment period on development of porcine PA blastocysts (PDF)

S4 Table Effect of FBS treatment during early IVC phase on early development and cell number of blastocysts in porcine PA embryos. (PDF)

S5 Table Effect of FBS and glutathione treatment during the early IVC phase on development of porcine PA embryos (PDF)

S6 Table Effect of FBS and glutathione treatment during the early IVC phase on ICM and TE proportion and cellular survival of porcine PA blastocysts (PDF)

S7 Table Effect of glutathione with hydrogen peroxide treatment during the early IVC phase on development of porcine PA embryos (PDF)

S8 Table Effect of FBS and hydrogen peroxide treatment during early IVC phase on development of porcine PA embryos (PDF)

S9 Table Effect of FBS and hydrogen peroxide treatment during early IVC phase on ICM and TE proportion and cellular survival of porcine PA blastocysts (PDF)

S10 Table Effect of FBS treatment during late IVC phase on early development in porcine PA embryos (PDF)

S11 Table Effect of FBS treatment during late IVC phase on ICM and TE proportion and cellular survival of porcine PA blastocysts (PDF)

S12 Table Effect of hydrogen peroxide treatment during late IVC phase on early development of porcine PA embryos (PDF)

S13 Table Effect of hydrogen peroxide with FBS treatment during late IVC phase on development of porcine PA embryos (PDF)

S14 Table Effect of hydrogen peroxide with FBS treatment during late IVC phase on ICM and TE proportion and cellular survival of porcine PA blastocysts (PDF)

S15 Table Effect of FBS with p38 MAPK and AKT inhibitors during the late IVC phase on development of porcine PA embryos (PDF)

S16 Table Effect of FBS with p38 MAPK and p-AKT inhibitors during late IVC phase on ICM and TE proportion and cellular survival of porcine PA blastocysts (PDF)

S17 Table Effect of FBS treatment during late IVC phase on development of porcine IVF embryos (PDF)

S18 Table Effect of FBS treatment during late IVC phase on ICM and TE proportion and cellular survival of porcine IVF blastocysts (PDF)

S19 Table Effect of FBS treatment during late IVC phase on development of porcine SCNT embryos (PDF)

S20 Table Effect of FBS treatment during late IVC phase on ICM and TE proportion and cellular survival of porcine SCNT blastocysts (PDF)
