# Supplementary figures and images for: Dual effect of fetal bovine serum on early development depends on stage-specific reactive oxygen species demands in pigs
Source: PLoS One. 2017 Apr 13;12(4):e0175427. doi: 10.1371/journal.pone.0175427 (PMC5391019; doi:10.1371/journal.pone.0175427)

**A**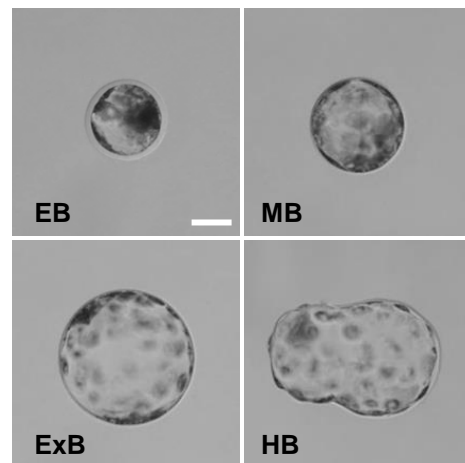**B**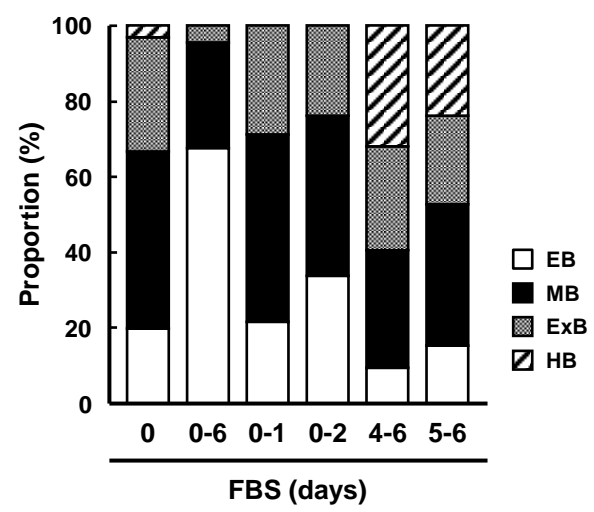

**Figure S1. Mun *et al.***

Supplement: S1 Fig — (A and B) Representative photographs (A) and proportion (B) of the blastocysts at four different embryonic stages (EB, early blastocyst; MB, mid-blastocyst; ExB, expanded blastocyst; HB, hatched or hatching blastocyst). (PDF) [file pone.0175427.s001.pdf]

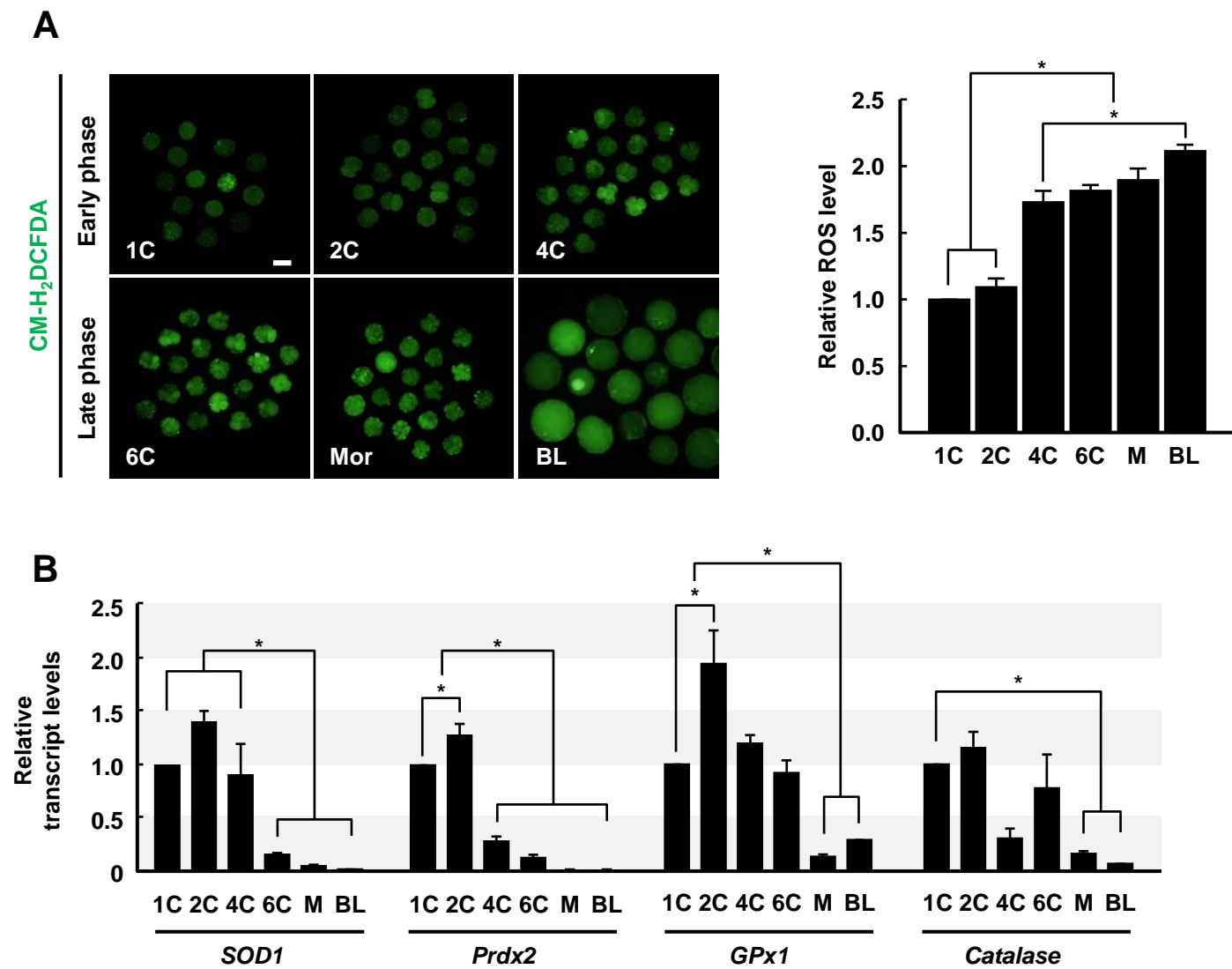

Figure S2. Mun *et al.*

Supplement: S2 Fig — (A) Fluorescence microscopy of 1-cell embryos to blastocysts treated with CM-H2DCFDA (left panel) and quantification of ROS levels (right panel) in the indicated groups. Bar = 100 μm. (B) qRT-PCR analysis of the relative abundance of Sod1, Prx II, Gpx1 and Bcl-xL transcript levels in each developmental stages. The data are from three independent experiments, and values represent the means ± SE (*P < 0.05). Abbreviations are 1C, 1-cell; 2C, 2-cell; 4C, 4-cell; 6C, 6-cell; M, morula; BL, blastocyst. (PDF) [file pone.0175427.s002.pdf]

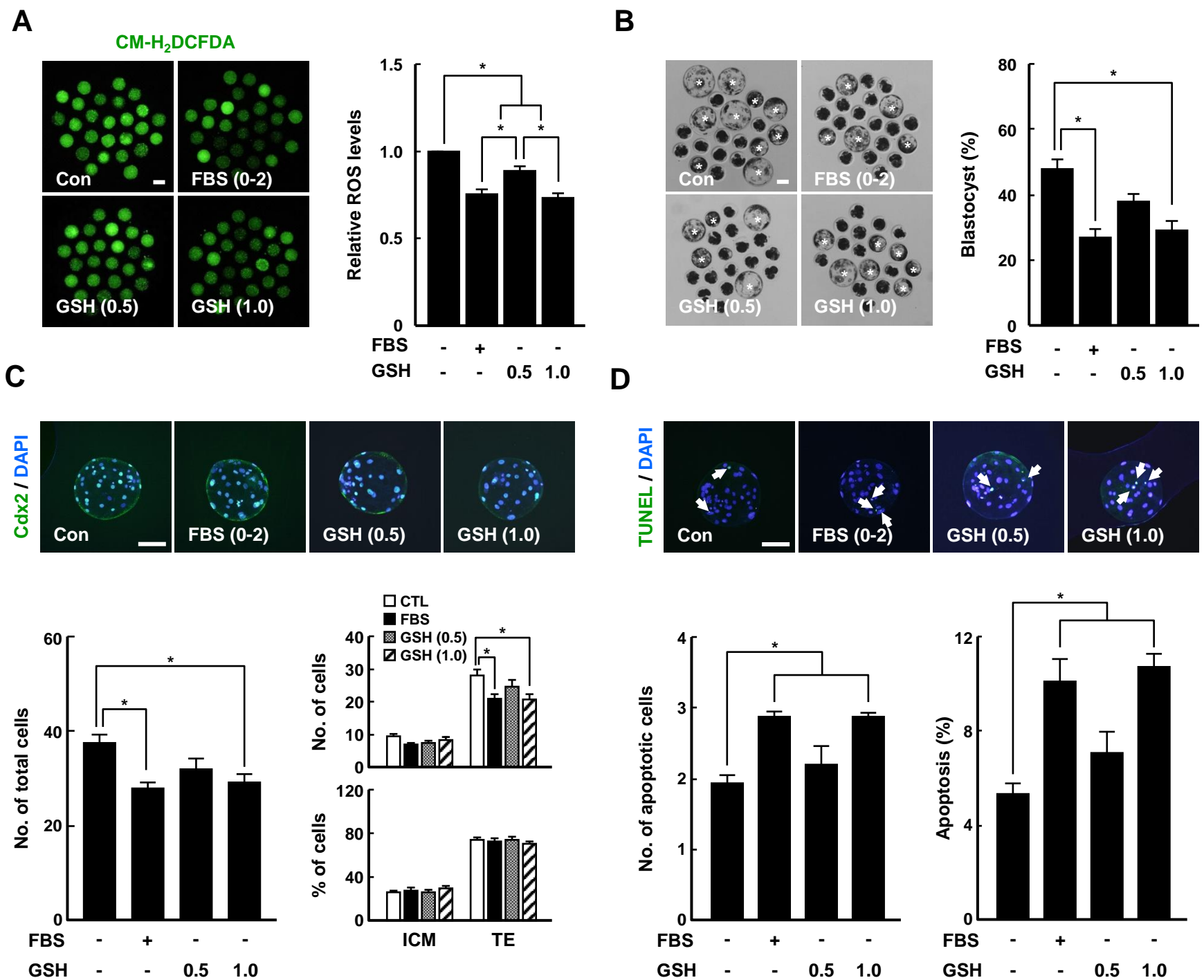

Figure S3. Mun et al.

Supplement: S3 Fig — (A) Fluorescence microscopy of 1-cell embryos treated with CM-H2DCFDA after 6 h of cultivation in the presence or absence of either FBS or GSH (0.5 and 1.0 μM) for 0–2 days of IVC (left panel) and quantification of ROS level (right panel) The data are from three independent experiments, and values represent the means ± SE (*P < 0.05). (B) Representative photographs of the developed blastocysts (white asterisks; left panel) within the indicated groups and quantification of blastocyst developmental rate (right panel) in the indicated groups. Bar = 100 μm. The data are from three independent experiments, and values represent the means ± SE (*P < 0.05). (C) Immunocytochemical analysis of Cdx2 using blastocysts developed under the indicated IVC conditions for 0–2 days of IVC and quantification of the total cell number (C; bottom left panel) and ICM/TE proportions (C; bottom right panel) in the indicated groups. Merged images (light green) between Cdx2 (green) and DAPI (blue) signals are shown (C; top panel). Bar = 100 μm (C). The data are from three independent experiments, and values represent the means ± SE (*P < 0.05; C). (D) Apoptosis detection analysis using blastocysts developed under the indicated IVC conditions for 0–2 days (D), and quantification of the number and proportion of apoptotic cells in the indicated groups (D; bottom panel). Merged images (light green) between TUNEL (green, white arrow) and DAPI (blue) signals are shown (D; top panel). Bar = 50 μm (D). The data are from three independent experiments, and values represent the means ± SE (*P < 0.05; D). (PDF) [file pone.0175427.s003.pdf]

**A**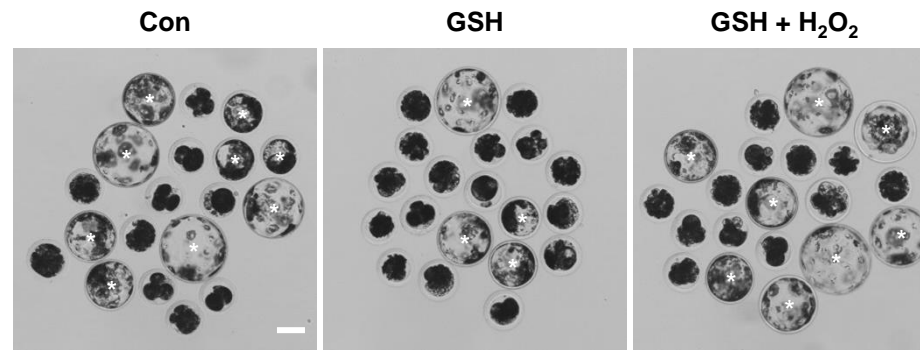**B**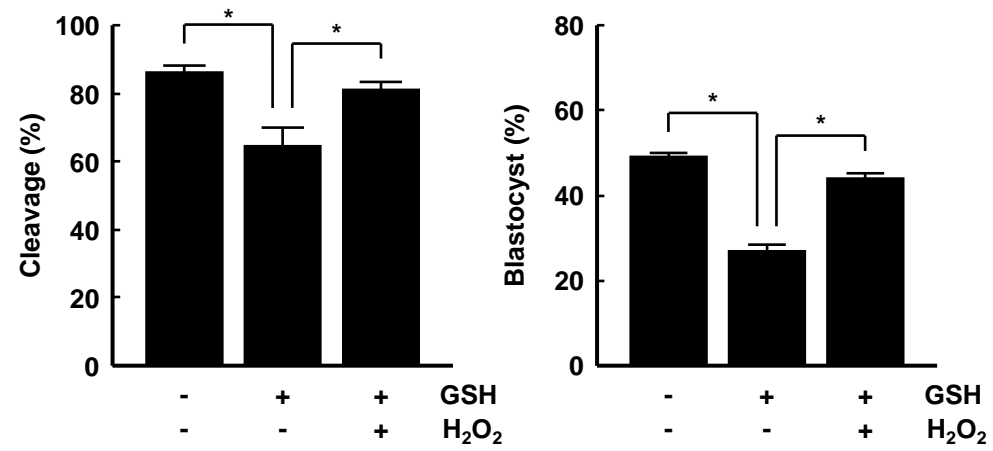

**Figure S4. Mun et al.**

Supplement: S4 Fig — (A and B) Representative photographs of blastocysts (white asterisks; A) developed in the presence or absence of 1.0 μM GSH and/or 0.1 mM hydrogen peroxide for 0–2 days of IVC and quantification of cleavage and blastocyst developmental rates in the indicated groups (B). Bar = 50 μm. (A). The data are from three independent experiments, and values represent the means ± SE (*P < 0.05; B). (PDF) [file pone.0175427.s004.pdf]
