# Supplementary material for: Dual effect of fetal bovine serum on early development depends on stage-specific reactive oxygen species demands in pigs
Source: PLoS One. 2017 Apr 13;12(4):e0175427. doi: 10.1371/journal.pone.0175427 (PMC5391019; doi:10.1371/journal.pone.0175427)
Supplement: S1 Table — (PDF) [file pone.0175427.s005.pdf]

Supplementary Table S1. Primer sequences used for semi-qRT-PCR and qRT-PCR

Gene	Primer sequences	GenBank accession no.	Product size (bp)
Cdx2	F: 5'-GGC AGC CAA GTG AAA ACC AG -3'<br>R: 5'-GCC TTT CTC CGA ATG GTG AT -3'	NM_001278769.1	119
Oct3/4	F: 5'-AGT GAG AGG CAA CCT GGA GA -3'<br>R: 5'-ACT GCT TGA TCG TTT GCC CT -3'	NM_174580.2	151
Bax	F: 5'-CTA CTT TGC CAG TAA ACT GG -3'<br>R: 5'-TCC CAA AGT AGG AGA GGA -3'	XM_005664710.1	158
Bcl-xl	F: 5'-AGG GCA TTC AGT GAC CTG AC -3'<br>R: 5'-TGG ATC CAA GGC TCT AGG TG-3'	NM_214285.1	242
SOD1	F: 5'-CTT CGA GCT GAA GGG AGA GAA-3'<br>R: 5'-CAA CGT GCC TCT CTT GAT CCT-3'	NM_001190422.1	185
Prdx2	F: 5'-CCT TCG CCA GAT CAC TGT CA-3'<br>R: 5'-TCC ACG TTG GGC TTG ATT GT-3'	NM_001244474.1	153
GPx1	F: 5'-TGG ACA TCA GGA AAA TGC CAA G-3'<br>R: 5'-GTG AGC ATT TGC GCC ATT CA-3'	NM_214201.1	127
Catalase	F: 5'-GCC GCC TAT TTG CCT ATC CT -3'<br>R: 5'-TTG GAG CCC CAC CTT GAT TG -3'	NM_214301.2	144
GAPDH	F: 5'-TCG GAG TGA ACG GAT TTG GC-3'<br>R: 5'-TGC CGT GGG TGG AAT CAT AC-3'	NM_001206359.1	147
