# Supplementary material for: Dual effect of fetal bovine serum on early development depends on stage-specific reactive oxygen species demands in pigs
Source: PLoS One. 2017 Apr 13;12(4):e0175427. doi: 10.1371/journal.pone.0175427 (PMC5391019; doi:10.1371/journal.pone.0175427)
Supplement: S2 Table — (PDF) [file pone.0175427.s006.pdf]

Supplementary Table S2. Effect of FBS supplementation timing on the developmental competence of porcine PA embryos

Groups	No. of embryos used	No. (%)* of embryos cleaved	No. (%)** of blastocysts developed	Total cell number of blastocyst (*n*)***
Control	120	103 (87.3±4.4)	60 (49.5±2.5)[b,c]	37.0±1.6[c] (45)
FBS (0–6)	209	166 (78.8±4.5)	31 (14.8±2.0)[f]	25.0±1.8[d] (31)
FBS (0–1)	115	96 (82.9±5.9)	48 (41.7±0.7)[c,d]	32.3±2.3[c,d] (33)
FBS (0–2)	106	75 (71.6±10.0)	33 (31.5±4.9)[d]	28.9±1.2[c,d] (33)
FBS (4–6)	124	105 (85.2±1.8)	79 (64.1±3.1)[a]	82.8±2.2[a] (45)
FBS (5–6)	108	92 (85.4±1.8)	59 (55.1±2.3)[a,b]	66.2±3.3[b] (59)

Data are the mean ± SEM, and values with different superscript letter within a column differ significantly ($p < 0.05$).
*Cleavage rate = (no. of embryos cleaved/no. of embryos used) ×100.
**Blastocyst development rate = (no. of blastocysts developed/no. of embryos used) ×100.
****n* = total no. of blastocysts used.
