# Supplementary material for: Dual effect of fetal bovine serum on early development depends on stage-specific reactive oxygen species demands in pigs
Source: PLoS One. 2017 Apr 13;12(4):e0175427. doi: 10.1371/journal.pone.0175427 (PMC5391019; doi:10.1371/journal.pone.0175427)
Supplement: S3 Table — (PDF) [file pone.0175427.s007.pdf]

Supplementary Table S3. Effect of FBS treatment period on development of porcine PA blastocysts

Groups	No. of blastocyst used	No. (%)* of blastocysts developed to the following stages at day 6 of IVC			
		EB	MB	ExB	HB
Control	60	12 (20.0±2.5)[b]	27 (46.8±7.4)	19 (30.1±7.0)[a]	2 (3.1±3.1)[b]
FBS (0–6)	31	20 (67.7±5.9)[a]	9 (27.9±5.9)	2 (4.4±2.9)[b]	0 (0.0±0.0)[b]
FBS (0–1)	48	10 (21.6±7.6)[b]	24 (49.6±11.2)	16 (28.9±4.2)[a]	0 (0.0±0.0)[b]
FBS (0–2)	33	11 (33.9±4.2)[b]	14 (42.2±1.3)	31 (23.9±3.2)[a]	0 (0.0±0.0)[b]
FBS (4–6)	79	7 (9.3±1.9)[b]	25 (31.0±3.0)	56 (27.8±3.1)[a]	25 (31.9±1.2)[a]
FBS (5–6)	59	9 (15.5±6.9)[b]	22 (37.2±1.0)	14 (23.3±6.5)[a]	14 (24.0±4.5)[a]

Data are the mean ± SEM, and values with different superscript letter within a column differ significantly ($p < 0.05$).

*Blastocysts development rate = (no. of blastocysts developed the indicated stage/no. of blastocysts used) ×100.

Abbreviations are EB, early blastocyst; MB, mid-blastocyst; ExB, expanded blastocyst; HB, hatching and hatched blastocysts.
