# Supplementary material for: Dual effect of fetal bovine serum on early development depends on stage-specific reactive oxygen species demands in pigs
Source: PLoS One. 2017 Apr 13;12(4):e0175427. doi: 10.1371/journal.pone.0175427 (PMC5391019; doi:10.1371/journal.pone.0175427)
Supplement: S4 Table — (PDF) [file pone.0175427.s008.pdf]

Supplementary Table S4. Effect of FBS treatment during early IVC phase on early development and cell number of blastocysts in porcine PA embryos

Groups	No. of embryos used	No. (%)[*] of embryos cleaved	No. (%)[**] of blastocysts developed	Total cell number of blastocyst (*n*)[***]
Control	132	109 (83.3±4.4)	66 (50.4±4.8)^{a}	35.8±0.2^{a} (54)
FBS (0–2)	119	91 (78.1±7.5)	39 (33.2±1.7)^{b}	29.1±1.1^{b} (33)

Data are the mean ± SEM, and values with different superscript letter within a column differ significantly ($p < 0.05$).
[*]Cleavage rate = (no. of embryos cleaved/no. of embryos used) ×100.
[**]Blastocyst development rate = (no. of blastocysts developed/no. of embryos used) ×100.
[***]*n* = total no. of blastocysts used.
