# Supplementary material for: Dual effect of fetal bovine serum on early development depends on stage-specific reactive oxygen species demands in pigs
Source: PLoS One. 2017 Apr 13;12(4):e0175427. doi: 10.1371/journal.pone.0175427 (PMC5391019; doi:10.1371/journal.pone.0175427)
Supplement: S5 Table — (PDF) [file pone.0175427.s009.pdf]

Supplementary Table S5. Effect of FBS and glutathione treatment during the early IVC phase on development of porcine PA embryos

| Groups | No. of embryos used | No. (%)* of embryos cleaved | No. (%)** of blastocyst developed |
|---|---|---|---|
| Control | 210 | 178 (84.9±3.5)[a] | 98 (47.6±3.8)[a] |
| FBS (0–2) | 234 | 148 (63.2±4.6)[b] | 64 (27.0±2.3)[b] |
| GSH (0.5 mM) | 242 | 200 (82.6±1.0)[a] | 92 (38.1±2.0)[a,b] |
| GSH (1.0 mM) | 238 | 152 (65.9±4.1)[b] | 70 (29.2±2.8)[b] |

Data are the mean ± SEM, and values with different superscript letter within a column differ significantly ($p < 0.05$).
*Cleavage rate = (no. of embryos cleaved/no. of embryos used) ×100.
**Blastocyst development rate = (no. of blastocysts developed/ no. of embryos used) ×100.
