# Supplementary material for: Dual effect of fetal bovine serum on early development depends on stage-specific reactive oxygen species demands in pigs
Source: PLoS One. 2017 Apr 13;12(4):e0175427. doi: 10.1371/journal.pone.0175427 (PMC5391019; doi:10.1371/journal.pone.0175427)
Supplement: S6 Table — (PDF) [file pone.0175427.s010.pdf]

Supplementary Table S6. Effect of FBS and glutathione treatment during the early IVC phase on ICM and TE proportion and cellular survival of porcine PA blastocysts

Groups	No. of blastocysts used	No. of cells			ICM (%)*	TE (%)**	No. of apoptotic cells (%)*** [*n*]****
		ICM	TE	Total			
Control	30	9.3±0.7	28.1±1.8[a]	37.4±1.8[a]	25.7±1.9	74.3±1.9	1.9±0.1[b] (5.3±0.4)[b] [33]
FBS (0–2)	30	6.9±0.4	20.9±1.5[b]	27.8±1.4[b]	27.3±2.8	72.7±2.8	2.9±0.1[a] (10.1±1.0)[a] [31]
GSH (0.5 mM)	30	7.4±0.6	24.6±2.1[a,b]	32.0±2.3[a,b]	25.7±2.4	74.3±2.4	2.2±0.3[a,b] (7.1±0.9)[a,b] [35]
GSH (1.0 mM)	30	8.3±0.8	20.7±1.6[b]	29.0±1.9[b]	29.3±2.0	70.7±2.0	2.9±0.1[a] (10.7±0.6)[a] [35]

Data are the mean ± SEM, and values with different superscript letter within a column differ significantly ($p < 0.05$).
*ICM proportion = (no. of ICM/no. of total cells in blastocyst) ×100.
**TE proportion = (no. of TE/no. of total cells in blastocyst) ×100.
***Apoptosis rate = (no. of apoptotic cells/no. of total cells in blastocyst) ×100.
****n = total no. of blastocysts used for TUNEL analysis.
