# Supplementary material for: Dual effect of fetal bovine serum on early development depends on stage-specific reactive oxygen species demands in pigs
Source: PLoS One. 2017 Apr 13;12(4):e0175427. doi: 10.1371/journal.pone.0175427 (PMC5391019; doi:10.1371/journal.pone.0175427)
Supplement: S7 Table — (PDF) [file pone.0175427.s011.pdf]

Supplementary Table S7. Effect of glutathione with hydrogen peroxide treatment during the early IVC phase on development of porcine PA embryos

Groups	No. of embryos used	No. (%)* of embryos cleaved	No. (%)** of blastocyst developed
Con	80	69 (86.3±1.7)[a]	39 (48.9±1.1)[a]
GSH (1.0 mM)	89	58 (64.5±5.4)[b]	24 (26.7±1.8)[b]
GSH (1.0 mM) + H_2O_2 (0.5 mM)	84	68 (81.0±2.4)[a]	37 (44.1±1.2)[a]

Data are the mean ± SEM, and values with different superscript letter within a column differ significantly ($p < 0.05$).
*Cleavage rate = (no. of embryos cleaved/no. of embryos used) ×100.
**Blastocyst development rate = (no. of blastocysts developed/ no. of embryos used) ×100.
