# Supplementary material for: Dual effect of fetal bovine serum on early development depends on stage-specific reactive oxygen species demands in pigs
Source: PLoS One. 2017 Apr 13;12(4):e0175427. doi: 10.1371/journal.pone.0175427 (PMC5391019; doi:10.1371/journal.pone.0175427)
Supplement: S8 Table — (PDF) [file pone.0175427.s012.pdf]

Supplementary Table S8. Effect of FBS and hydrogen peroxide treatment during early IVC phase on development of porcine PA embryos

Groups	No. of embryos used	No. (%)* of embryos cleaved	No. (%)** of blastocysts developed
Control	174	146 (83.9±2.1)	84 (48.2±1.1)[a]
FBS (0–2)	238	154 (69.9±9.2)	62 (24.7±2.9)[b]
H_2O_2 (0.5 mM)	172	154 (89.5±2.0)	80 (46.6±2.6)[a]
FBS (0–2) + H_2O_2 (0.5 mM)	182	134 (73.8±6.4)	72 (39.5±0.6)[a]

Data are the mean ± SEM, and values with different superscript letter within a column differ significantly ($p < 0.05$).
*Cleavage rate = (no. of embryos cleaved/no. of embryos used) ×100.
**Blastocyst development rate = (no. of blastocysts developed/no. of embryos used) ×100.
