# Supplementary material for: Dual effect of fetal bovine serum on early development depends on stage-specific reactive oxygen species demands in pigs
Source: PLoS One. 2017 Apr 13;12(4):e0175427. doi: 10.1371/journal.pone.0175427 (PMC5391019; doi:10.1371/journal.pone.0175427)
Supplement: S10 Table — (PDF) [file pone.0175427.s014.pdf]

Supplementary Table S10. Effect of FBS treatment during late IVC phase on early development in porcine PA embryos

Groups	No. of embryos used	No. (%)* of embryos cleaved	No. (%)** of blastocysts developed	Total cell number of blastocyst (n)***
Control	266	226 (84.9±2.6)	138 (52.0±2.3)[b]	35.8±0.9[b] (50)
FBS (4–6)	281	227 (80.4±3.3)	171 (60.9±2.2)[a]	82.5±4.5[a] (48)

Data are the mean ± SEM, and values with different superscript letter within a column differ significantly ($p < 0.05$).
*Cleavage rate = (no. of embryos cleaved/no. of embryos used) ×100.
**Blastocyst development rate = (no. of blastocysts developed/no. of embryos used) ×100.
***n = total no. of blastocysts used.
