# Supplementary material for: Dual effect of fetal bovine serum on early development depends on stage-specific reactive oxygen species demands in pigs
Source: PLoS One. 2017 Apr 13;12(4):e0175427. doi: 10.1371/journal.pone.0175427 (PMC5391019; doi:10.1371/journal.pone.0175427)
Supplement: S11 Table — (PDF) [file pone.0175427.s015.pdf]

Supplementary Table S11. Effect of FBS treatment during late IVC phase on ICM and TE proportion and cellular survival of porcine PA blastocysts

Groups	No. of blastocysts used	No. of cells			ICM (%)*	TE (%)**	No. of apoptotic cells (%)*** [*n*]****
		ICM	TE	Total			
Con	30	11.8±1.6	29.2±4.9[b]	41.0±5.3[b]	33.6±3.1[a]	66.4±3.1[b]	2.6±0.1[a] (7.2±0.1)[a] [41]
FBS (4–6)	30	12.6±1.3	91.5±11.4[a]	104.1±10.9[a]	12.4±4.6[b]	87.6±4.6[a]	1.0±0.1[b] (1.0±0.1)[b] [35]

Data are the mean ± SEM, and values with different superscript letter within a column differ significantly ($p < 0.05$).
*ICM proportion = (no. of ICM/no. of total cells in blastocyst) ×100.
**TE proportion = (no. of TE/no. of total cells in blastocyst) ×100.
***Apoptosis rate = (no. of apoptotic cells/no. of total cells in blastocyst) ×100.
****n = total no. of blastocysts used for TUNEL analysis.
