# Supplementary material for: Dual effect of fetal bovine serum on early development depends on stage-specific reactive oxygen species demands in pigs
Source: PLoS One. 2017 Apr 13;12(4):e0175427. doi: 10.1371/journal.pone.0175427 (PMC5391019; doi:10.1371/journal.pone.0175427)
Supplement: S12 Table — (PDF) [file pone.0175427.s016.pdf]

Supplementary Table S12. Effect of hydrogen peroxide treatment during late IVC phase on early development of porcine PA embryos

H_2O_2 (mM)	No. of embryos used	No. (%)[*] of embryos cleaved	No. (%)[**] of blastocysts developed
0	109	87 (80.1±1.0)	59 (55.9±4.7)[a]
0.1	110	88 (80.2±1.0)	49 (44.4±3.5)[a,b]
0.5	108	86 (79.9±0.8)	38 (34.0±6.0)[b]

Data are the mean ± SEM, and values with different superscript letter within a column differ significantly ($p < 0.05$).
[*]Cleavage rate = (no. of embryos cleaved/no. of embryos used)×100.
[**]Blastocyst development rate = (no. of blastocysts developed/ no. of embryos used)×100.
