# Supplementary material for: Dual effect of fetal bovine serum on early development depends on stage-specific reactive oxygen species demands in pigs
Source: PLoS One. 2017 Apr 13;12(4):e0175427. doi: 10.1371/journal.pone.0175427 (PMC5391019; doi:10.1371/journal.pone.0175427)
Supplement: S13 Table — (PDF) [file pone.0175427.s017.pdf]

Supplementary Table S13. Effect of hydrogen peroxide with FBS treatment during late IVC phase on development of porcine PA embryos

Groups	No. of embryos used	No. (%)* of embryos cleaved	No. (%)** of blastocysts developed
Control	314	256 (81.4±1.1)	136 (43.2±3.1)[a]
H_2O_2 (0.5 mM)	316	254 (80.2±1.3)	60 (18.9±2.7)[b]
FBS (4–6) + H_2O_2 (0.5 mM)	314	252 (80.2±0.8)	100 (31.9±3.9)[a]

Data are the mean ± SEM, and values with different superscript letter within a column differ significantly ($p < 0.05$).
*Cleavage rate = (no. of embryos cleaved/no. of embryos used) ×100.
**Blastocyst development rate = (no. of blastocysts developed/no. of embryos used) ×100.
