# Supplementary material for: Dual effect of fetal bovine serum on early development depends on stage-specific reactive oxygen species demands in pigs
Source: PLoS One. 2017 Apr 13;12(4):e0175427. doi: 10.1371/journal.pone.0175427 (PMC5391019; doi:10.1371/journal.pone.0175427)
Supplement: S15 Table — (PDF) [file pone.0175427.s019.pdf]

Supplementary Table S15. Effect of FBS with p38 MAPK and AKT inhibitors during the late IVC phase on development of porcine PA embryos

Groups	No. of embryos used	No. (%)[*] of embryos cleaved	No. (%)[**] of blastocyst developed
Control	144	121 (83.7±1.4)	69 (47.5±4.2)[b]
FBS (4–6)	129	109 (84.6±1.6)	82 (63.7±3.5)[a]
FBS (4–6) + SB203580	133	111 (83.1±1.7)	63 (46.1±3.0)[b]
FBS (4–6) + LY294002	155	130 (83.7±1.5)	68 (43.3±3.3)[b]

Data are the mean ± SEM, and values with different superscript letter within a column differ significantly ($p < 0.05$).
[*]Cleavage rate = (no. of embryos cleaved/no. of embryos used) ×100.
[**]Blastocyst development rate = (no. of blastocysts developed/no. of embryos used) ×100.
