# Supplementary material for: Dual effect of fetal bovine serum on early development depends on stage-specific reactive oxygen species demands in pigs
Source: PLoS One. 2017 Apr 13;12(4):e0175427. doi: 10.1371/journal.pone.0175427 (PMC5391019; doi:10.1371/journal.pone.0175427)
Supplement: S16 Table — (PDF) [file pone.0175427.s020.pdf]

Supplementary Table S16. Effect of FBS with p38 MAPK and p-AKT inhibitors during late IVC phase on ICM and TE proportion and cellular survival of porcine PA blastocysts

Groups	No. of blastocysts used	No. of cells			ICM (%)*	TE (%)**	No. of apoptotic cells (%)*** [*n*]****
		ICM	TE	Total			
Control	30	11.2±0.9	30.8±1.0[d]	42.0±0.3[d]	30.8±2.4[a]	69.2±2.4[c]	2.8±0.2[a] (6.8±0.2)[a] [30]
FBS (4–6)	30	12.4±0.6	94.3±3.2[a]	107.7±3.8[a]	11.8±0.9[c]	88.2±0.9[a]	1.6±0.2[b] (2.2±0.1)[b] [30]
FBS (4–6) + SB203580	30	10.5±0.7	46.4±2.5[c]	56.9±2.4[c]	19.3±1.6[b]	80.7±1.6[b]	1.8±0.1[b] (3.0±0.3)[b] [30]
FBS (4–6) + LY294002	33	13.1±3.9	70.1±2.2[b]	83.1±1.7[b]	16.8±1.5[b,c]	83.2±1.5[a,b]	1.9±0.1[b] (3.6±0.5)[b] [30]

Data are the mean ± SEM, and values with different superscript letter within a column differ significantly ($p < 0.05$).
*ICM proportion = (no. of ICM/no. of total cells in blastocyst) ×100.
**TE proportion = (no. of TE/no. of total cells in blastocyst) ×100.
***Apoptosis rate = (no. of apoptotic cells/no. of total cells in blastocyst) ×100.
**** *n* = total no. of blastocysts used for TUNEL analysis.
