# Supplementary material for: Dual effect of fetal bovine serum on early development depends on stage-specific reactive oxygen species demands in pigs
Source: PLoS One. 2017 Apr 13;12(4):e0175427. doi: 10.1371/journal.pone.0175427 (PMC5391019; doi:10.1371/journal.pone.0175427)
Supplement: S17 Table — (PDF) [file pone.0175427.s021.pdf]

Supplementary Table S17. Effect of FBS treatment during late IVC phase on development of porcine IVF embryos

Groups	No. of embryos used	No. (%)* of embryos cleaved	No. (%)** of blastocyst developed	No. (%)*** of HB developed
Control	223	178 (79.9±2.7)	80 (35.5±3.2)^{b}	7 (7.1±4.1)^{b}
FBS (4–6)	223	180 (80.8±2.2)	110 (48.9±4.1)^{a}	36 (33.3±7.9)^{a}

Data are the mean ± SEM, and values with different superscript letter within a column differ significantly ($p < 0.05$).
*Cleavage rate = (no. of embryos cleaved/no. of embryos used) ×100.
**Blastocyst development rate = (no. of blastocysts developed/no. of embryos used) ×100.
**HB development rate = (no. of HB blastocysts developed/ no. of embryos developed) ×100.
Abbreviation is HB, hatching and hatched blastocyst.
