# Supplementary material for: Dual effect of fetal bovine serum on early development depends on stage-specific reactive oxygen species demands in pigs
Source: PLoS One. 2017 Apr 13;12(4):e0175427. doi: 10.1371/journal.pone.0175427 (PMC5391019; doi:10.1371/journal.pone.0175427)
Supplement: S19 Table — (PDF) [file pone.0175427.s023.pdf]

Supplementary Table S19. Effect of FBS treatment during late IVC phase on development of porcine SCNT embryos

Groups	No. of embryos used	No. (%)* of embryos cleaved	No. (%)** of blastocyst developed
Control	203	171 (84.0±1.2)	69 (34.3±0.8)[b]
FBS (4–6)	184	152 (82.7±0.1)	91 (49.2±2.1)[a]

Data are the mean ± SEM, and values with different superscript letter within a column differ significantly ($p < 0.05$).
*Cleavage rate = (no. of embryos cleaved/no. of embryos used) ×100.
**Blastocyst development rate = (no. of blastocysts developed/ no. of embryos used) ×100.
